# Facial expression in humans as a measure of empathy towards farm animals in pain

**DOI:** 10.1371/journal.pone.0247808

**Published:** 2021-03-01

**Authors:** Lexis H. Ly, Daniel M. Weary

**Affiliations:** Animal Welfare Program, Faculty of Land and Food Systems, University of British Columbia, Vancouver, BC, Canada; Virginia Commonwealth University, UNITED STATES

## Abstract

People often express concern for the welfare of farm animals, but research on this topic has relied upon self-report. Facial expressions provide a quantifiable measure of emotional response that may be less susceptible to social desirability bias and other issues associated with self-report. Viewing other humans in pain elicits facial expressions indicative of empathy. Here we provide the first evidence that this measure can also be used to assess human empathetic responses towards farm animals, showing that facial expressions respond reliably when participants view videos of farm animals undergoing painful procedures. Participants (*n* = 30) were asked to watch publicly sourced video clips of cows and pigs undergoing common management procedures (e.g. disbudding, castration, tail docking) and control videos (e.g. being lightly restrained, standing). Participants provided their subjective rating of the intensity of 5 negative emotions (pain, sadness, anger, fear, disgust) on an 11-point Likert scale. Videos of the participants (watching the animals) were scored for intensity of unpleasantness of the participants’ facial expression (also on an 11-point Likert scale) by a trained observer who was blind to treatment. Participants showed more intense facial expressions while viewing painful procedures versus control procedures (mean ± SE Likert; 2.4 ± 0.08 versus 0.6 ± 0.17). Participants who reported more intense negative responses also showed stronger facial expressions (slope ± SE = 0.4 ± 0.04). Both the self-reported and facial measures varied with species and procedure witnessed. These results indicate that facial expressions can be used to assess human-animal empathy.

## 1. Introduction

Empathy has long been known to play a role in interpersonal relationships [1]. Empathy is expressed towards other humans [2], and also to non-human animals [3], through a variety of physiological and behavioural responses. Empathy shapes the way humans view and care for animals [4]. For example, farmer empathy affects attitudes towards animals, and in turn affects the welfare of animals in their care [5]. Empathy of members of the general public impacts their attitudes towards animal use [6].

The Perception-Action Model (PAM) of empathy provides a basis for understanding vicarious facial expressions. This model states that perception of someone’s emotional state activates a similar state in the observer [1]. Empathy is an automatic, unconscious response that causes the observer to experience emotions similar to those that they perceive are experienced by others [7]. The stimuli for the PAM of empathy can be another person, animal, or even broader entity (e.g. ‘the earth’) [2]. The literature regarding the PAM of empathy has been most developed for neurological changes associated with empathetic response in humans. Similar neural mechanisms are present when experiencing firsthand pain or disgust compared to viewing facial expressions of these same emotions [8]. If an emotional stimulus is strong enough, it can produce similar physiological and behavioural responses in the observer [9]. For example, when viewing facial expressions of emotions in humans, participants often show vicarious facial expressions of the same emotions [10].

There is evidence that the PAM of empathy can be applied when the subject is a human and the object is an animal—particularly when viewing animals in pain. Angantyr et al. [11] found that people self-reported at least the same level of empathy for a puppy as they did a human baby when asked to rate their feeling of emotions related to empathy. Similar to studies on human-human empathy, human-animal empathy has been assessed through neurological (e.g. fMRI) and physiological means (e.g. phasic skin conductance, heart rate). These studies have found that humans show similar responses towards humans and animals in victimizing scenarios [3, 12, 13], suggesting that the mechanisms involved in the automatic process of human-human empathy are also relevant in the process of human-animal empathy. The human-animal empathetic response has been assessed through self-reported measures, neurological response, and some physiological responses, but to our knowledge has yet to be assessed through facial expressions. Studies have described facial expressions of basic emotions in humans through specific muscle movements of the face [14–16]. Non-verbal behavioural responses are more difficult to feign compared to verbal reports of emotion [17], as verbal self-report is a conscious cognitive interpretation of one’s emotional state, rather than an autonomic one [18]. The use of multiple measures of emotional response can provide greater validity and help understand differences between measures [16]. In research regarding human-human empathy, facial expression is a reliable measure of empathetic response [9], suggesting that this may also be useful in assessing human-animal empathy given similarities in the empathetic process [11].

None of the aforementioned studies on human-animal empathy assess empathy toward farm animals. There is growing concern for the welfare of animals on farms, particularly in relation to common farm management practices that affect animal welfare [19–22]. The existing literature on attitudes towards farm animals only involves the use of self-reported measures, a method which is subjected to bias [23]. The PAM of empathy emphasizes the effect of “similarity”, such as human appearance or phylogenetic similarity; this has been assessed by comparing empathetic response toward species of varying similarity to humans such as primates, birds, and companion animals [2, 3]. Although morphologically dissimilar to humans, we have a close historical relationship to these animals such that their pain may evoke empathetic responses in humans, measurable by facial expression. A better understanding of empathetic responses to pain in these animals is also of practical relevance given the number of animals (and painful procedures) involved, and because of the growing disconnect between modern urban dwellers and agricultural production [24].

The objective of the present study was to assess the use of facial expression to assess empathetic response toward farm animals undergoing common painful procedures. We predicted that participants would show more intense expressions of negative emotion when viewing the painful procedures versus ‘control’ videos of animals experiencing neutral situations or non-painful procedures. We also predicted that participant facial expressions of negative emotion would be positively correlated with self-reported measures of empathy. Finally, we examined how top-down processes such as gender and trait empathy, and bottom-up processes such as species viewed and the video stimuli shown, affected the intensity of facial expression and self-reported response.

## 2. Materials and methods

### 2.1. Participants

We assessed responses of 30 undergraduate students from the University of British Columbia (UBC; Vancouver, Canada) in August 2019. Our sample size was based on three previous studies [18, 25, 26] that used 34, 30 and 44 participants, respectively. These studies assessed participant emotional responses to facial expressions of emotions in humans and dogs, to videos of humans in pain, and to humans showing happy and angry emotions. These previous studies did not report effect size and variation in a way that allowed us to perform a formal power analysis, but all three studies were able to assess treatment effects with the sample sizes used.

Undergraduate students at the University of British Columbia (UBC) were recruited through email, class announcements, UBC-related Facebook groups and online forums, and given a $10 gift card. Participants were required to be at least 18 years old to consent to this study; demographics are described in Table 1. Participants reported a low knowledge of animal production, with the mean (± SD) Likert score of 3.6 (± 2.2) on an 11-point Likert scale. All procedures were approved by the UBC Behavioral Ethics Review Board (Ethics ID: H19-01683).

**Table 1:** Demographics of the interview participants (n = 30).

Demographic Factor		n
Gender	Male	16
	Female	14
Diet	Meat-related restrictions (e.g. vegetarian, vegan, pescetarian, do not eat beef or pork)	8
Faculty of study	Science	8
	Land and Food Systems	8
	Engineering	7
	Arts	6
	Forestry	1
Last farm visit	Never visited a farm	1
	Within their lifetime	14
	Within the last year	8
	Within the last six months	4
	Within the last month	2
	Within the last week	1
		**mean (± SD)**
Age		20.4 (± 1.3)

### 2.2. Survey

Before the interview, participants completed the Interpersonal Reactivity Index (IRI), commonly used to measure empathy [6, 27, 28]. Previous studies have shown that two of the subscales—Empathetic Concern (ability to feel compassion for others in negative experiences) and Personal Distress (distress caused by others’ negative experiences)—positively correlate with attitudes towards animals [6, 28], so we tested the relationship between these subscales and both facial expression and self-reported responses to the videos.

### 2.3. Interview conditions

Participants completed the video assessment individually in a private office on the university campus. Participants faced a computer monitor (ViewSonic VA2246mh-LED) and an attached webcam (Logitech C920S Pro). The interviewer sat opposite the participant behind the monitor so their face was not visible to the participant. The interviewer played the videos from a laptop, which appeared on the participant’s monitor in full-screen mode. A tone was played one second before and after each video clip (to later synch responses with the clip start and end) but otherwise no sound from the video clip was played. The head and shoulders of the participant were video recorded while the clips were played.

### 2.4. Interview

Participants consented to being video recorded, but were not told of our intention to analyse their facial expressions. The interviewer read a short excerpt which briefly described the procedure (see S1 File for excerpts). The participant then viewed the 10 second clip showing the procedure (video playlist can be found here). Each participant was shown 10 videos in total: three painful procedures on piglets (tail docking, teeth clipping, surgical castration), three others on cattle (branding, disbudding, banding castration), plus two control videos for each species. Control videos were of the animal standing or being restrained without any procedure occurring. All videos shown were sourced from YouTube, and were selected on the basis of video quality and as being similar to common practice in cattle and pig farming in North America. All videos were collected in compliance with YouTube’s Terms and Conditions. A total of 20 videos were chosen selected (i.e., two examples of each procedure and control video), but each participant was only shown one of the two examples. The order in which the videos were shown was assigned using Latin square design.

Immediately after viewing each video, participants were asked whether a particular emotion described how they felt towards the video based on a list of seven basic emotions (surprise, happiness, sadness, pain, anger, fear, disgust). Participants were then asked to rate the intensity of their emotions on an 11-point Likert scale (0 = not intense at all, 10 = very intense; based on [11, 29]. Participants were allowed as much time as they desired to answer the questions related to one video before the next video was shown. The time between successive video presentations was not measured, but the total session (including viewing and responding to all videos) lasted approximately 20 minutes for each participant.

### 2.5. Video scoring

The current study employs a method called thin slicing, which requires little training to reliably code temporary affect [30, 31]. Thin slicing uses a short clip (a “thin slice”) of expressive behaviour sampled from a larger behavioural stream [32]. The thin slice of expressive behaviour can be shown to naïve observers who then score the clip for affective state [30]. These scores can then be compared to the participants’ own self-report [32]. In the current study, thin slicing was used to assess facial expression of negative emotions.

A 14 second clip (that included the two seconds before and after the video stimuli) of each participants watching each video was saved as a separate file using a non-identifiable file name. Two observers, both blind to the video stimuli being played, scored participants’ facial expressions using an 11-point scale. Observers were asked to score negative facial expressions (e.g. pain, disgust, sadness, fear, anger) as outlined in previous studies of these emotions [15, 16, 33]. Criteria for unpleasantness included facial motions such as lowering of the brows, cheek raising, eyelid tightening, nose wrinkling, chin raising, lip puckering, frowning, lip tightening, jaw dropping, mouth stretching. Observers were asked to rate each video for the intensity of unpleasantness of the facial expression, from 0 for not unpleasant at all to10 for very unpleasant. Observers were trained using 10 participants whose videos were taken during practice interviews. Scores were discussed between observers and the observers re-scored the practice videos. Following agreement, the observers independently scored five randomly selected participants from the current sample, and then compared scores to ensure agreeability. After final agreement, all videos were scored independently by each observer. Inter-observer agreement was calculated through a two-way intraclass correlation across all videos and the resulting coefficient was 0.92.

### 2.6. Statistical analysis

An error occurred in recording one participant; for this participant data were missing for facial expression in response to one pig control video (PN1). For facial expression analysis, scores were averaged across both observers over all videos. Self-reported ratings for pain, disgust, anger, fear, sadness were averaged for each video to create one score of “self-reported intensity of negative emotion”. We chose to average these five emotions because 1) they are known to be negatively valenced [34] and 2) we had a priori predictions based on negative emotional response, which led us to score facial expression response on a scale of negative emotion. Facial expression and self-reported emotional responses were negatively skewed; data were normalized before analysis using a square-root transformation.

For both facial expression and self-reported emotional response, the effect of treatment (i.e. painful vs. control videos), species (pig vs. cow), the order in which the videos were presented, participant gender, diet, Empathetic Concern score and Personal Distress score, as well as all first order interactions, were tested using a mixed model, specifying participant identity as random effect and using compound symmetry as the covariance structure selected on the basis of model fit (as assessed using the AIC).

The association between facial expression and self-report was also assessed using a mixed model, testing the effect on facial expression of self-report and the order in which the videos were presented, specifying participant identity as random effect and using compound symmetry as the covariance structure selected on the basis of model fit (as assessed using the AIC).

## 3. Results & discussion

### 3.1. Emotional response towards procedural videos versus control videos

**Fig 1:**
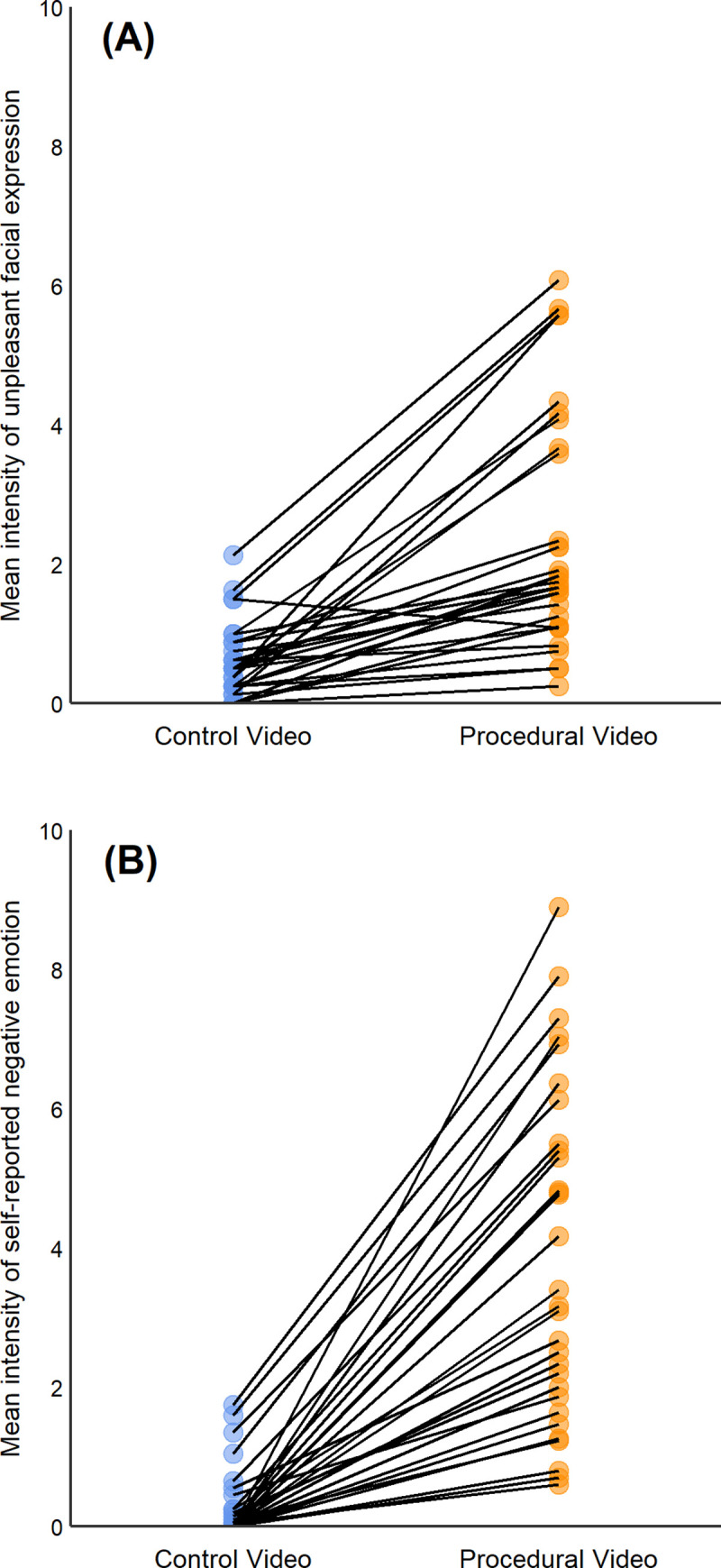
Mean emotional response for each participant when watching control videos versus procedural videos. Data are illustrated for both the intensity of facial expression (A), and self-reported emotion (B). Control videos show cows and pigs in neutral scenarios (e.g. standing, being held) and procedural videos show cows and pigs undergoing painful procedures (e.g. disbudding, castration). The individual lines connect the two means illustrated for each participant (*n* = 30).

Videos of participants who consented for these to be included in the publication can be viewed in the linked playlist. Fig 1 shows that participant responses varied for both facial expression and self-reported responses.

The intensity of unpleasant facial expressions was higher in response to the procedural versus control videos (F_1, 257_ = 131.4, p<0.0001); 29 of the 30 participants showed an increase in intensity of unpleasant facial expression when viewing a video showing a painful procedure (Fig 1A). We found no effect of species (cow vs. pig) or interaction between species and the main effect of pain vs. control video (all comparisons p>0.1; Table 1). There was no effect of the order in which videos were shown, or of participant gender, diet, or empathy score on facial expression scores, and no interactions between these factors. There was, however, an interaction with both Empathetic Concern and Personal Distress and pain versus control videos (Empathetic Concern F_1,257_ = 8.1, p = 0.005; Personal Distress F_1, 257_ = 7.8, p = 0.006). Given these interactions, we we-ran this model separately by painful or control video, and found that increased Empathetic Concern and Personal Distress were associated with decreased intensity of negative facial expression response toward control videos (Empathetic Concern slope ± SE = -0.2 ± 0.1; Personal Distress slope ± SE = -0.2 ± 0.2) and increased negative facial expression response toward procedural videos (Empathetic Concern slope ± SE = 0.3 ± 0.2; Personal Distress slope ± SE = 0.3 ± 0.2), although these individual effects were not significant (p>0.05 for all comparisons).

Similar to facial expression scores, there was considerable variation between individuals in self-reported emotional responses (Fig 1B). The intensity of the self-reported negative emotions was higher in response to the procedural videos (F_1, 257 =_ 661.0, p<0.0001), a pattern apparent for all 30 of the participants. There was an effect of species on self-reported response, with participants reporting higher negative emotional responses toward cow versus pig videos (Table 2; F_1, 257_ = 8.3, p = 0.004). Empathetic Concern also had an effect on self-reported responses—an increase in one-unit of Empathetic Concern increased self-reported emotional response toward procedural videos by 0.5 of a Likert score (± 0.2; F_1,25_ = 5.88, p = 0.02). No other demographic effects were detected.

**Table 2:** Mean ± SE facial expression and self-reported response of participants (*n* = 30) in relation to the species of animal shown in the video.

	Facial Expression		Self-Report	
Species	Procedural	Control	Procedural	Control
Pig	2.4 ± 0.23	0.5 ± 0.10	3.6 ± 0.28	0.1 ± 0.05
Cow	2.4 ± 0.25	0.7 ± 0.12	4.1 ± 0.30	0.4 ± 0.14

Responses are shown separately for videos showing a painful procedure versus control videos showing the animals restrained or standing.

When participants showed more intense facial expressions of emotion their self-reported negative emotions were also higher. For every one-unit increase in self-reported response, intensity of facial expression response increased by 0.4 (±0.04; F_1,288_ = 11.69, p<0.0001).

### 3.2. Differences between individual video stimuli

**Fig 2:**
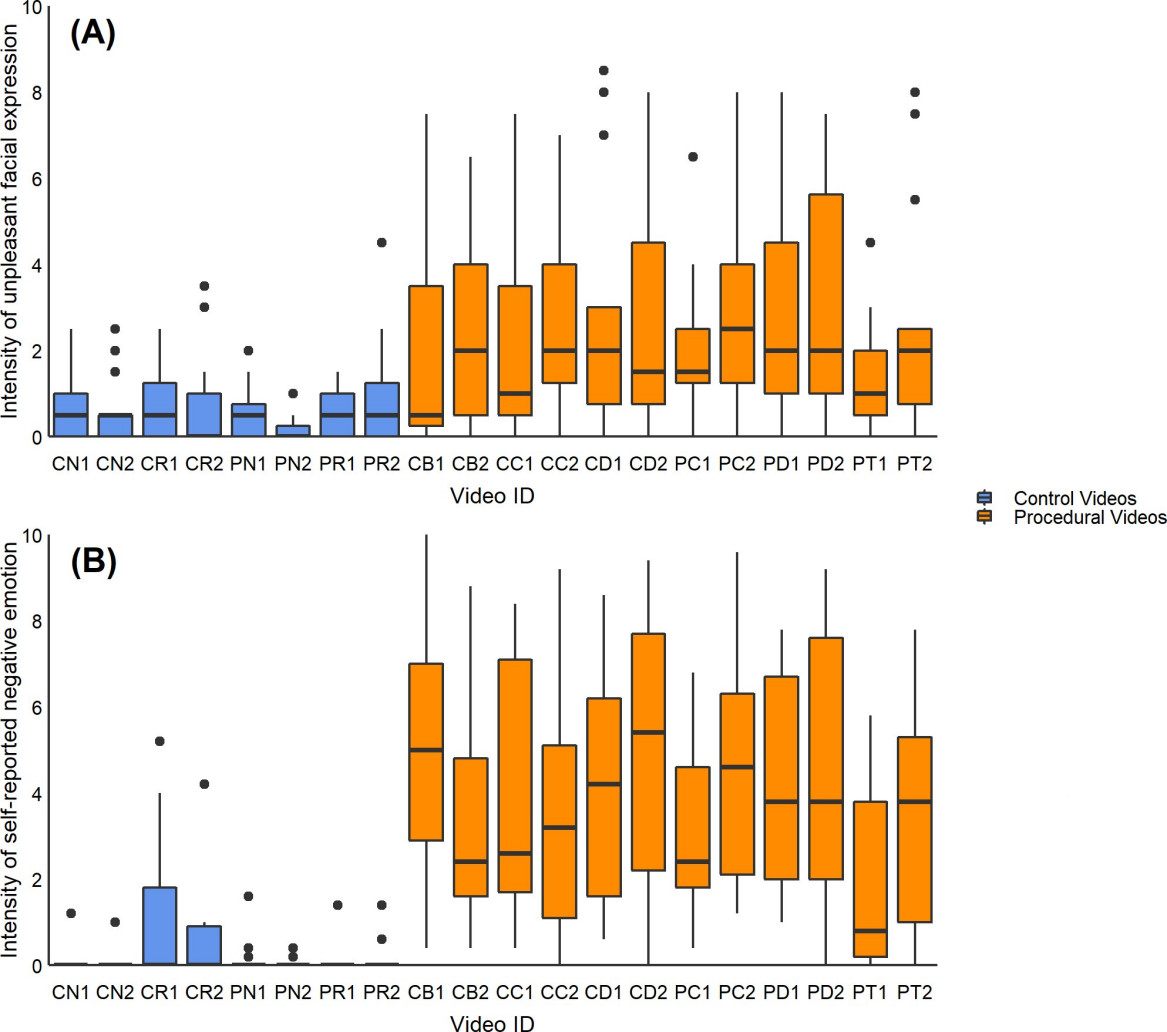
Intensity of emotional response towards each video procedure and version (PD2 n = 14, all others *n* = 15). Data are illustrated for both the intensity of facial expression (A), and self-reported emotion (B). Video ID is labelled by procedure (CB = cow branding, CC = cow castration, CD = cow disbudding, PC = pig castration, PD = pig tail docking, PT = pig teeth clipping, CN = cow neutral (standing), CR = cow restraint, PN = pig neutral (standing), PR = pig restraint). The numbers 1 or 2 indicate which of the 2 sets of videos were shown. Each participant was shown one of the two sets (1 or 2). The central line in the boxplots shows the median, the limits of the box show the 1^st^ and 3^rd^ quartiles, and the whiskers show the 10^th^ and 90^th^ percentiles.

We did not have *a priori* predictions about differences in response to the 20 specific videos shown, so this data is shown only for descriptive purposes in Fig 2A and 2B. There was considerable variation among videos, including to different versions showing the same procedure (most notably the cattle branding, cattle castration and piglet tail docking videos). These differences suggest some effect of differences in the composition of the video stimuli. It is possible that contextual factors (e.g. environment, farm workers) may have affected participant response (see [35]). However, despite the variability between video versions and procedures, the median facial response for all but one procedural video was greater than the medians for control videos. This result adds some generality to the finding that facial expressions are stronger towards procedural videos.

Similarly, self-reported emotional responses varied among videos, such as between the two versions of the cattle branding, piglet castration and piglet tail docking videos. The median self-reported emotional responses for all control videos was 0, indicating that the participants’ conscious interpretations of the control videos were not unpleasant.

## 4. Discussion

Previous research has investigated human-animal empathy through self-report and physiological response [11, 12], but to our knowledge the current study is the first to assess human-animal empathy using facial expression. Further, no previous studies on human-animal empathy have assessed human emotional responses toward farm animals. Recent survey work has suggested that the citizens are increasingly concerned for the welfare of farm animals, including how this is affected by common management procedures [20, 36, 37]. Our study provides first evidence that facial expression can be used as a valid measure of empathetic response of humans towards farm animals undergoing painful procedures.

Our primary objective was to determine the extent to which facial expressions can serve as an indicator of emotional responses in people witnessing common but painful farm procedures. The PAM of empathy states that the degree of neural activation within the subject and object may vary, so the representation through behaviour may not be present if the activation is not strong enough [2]. However, should the object be strong enough, the subject’s facial expression functions as a reflection of the person’s own experience of the emotion [9]. Westbury & Neumann [3] used phasic skin conductance response, and Prguda & Neumann [12] used skin conductance and heart rate change to assess empathetic response towards animals, while other studies used survey methods [38, 39]. Our study similarly found that humans showed empathetic response toward farm animals in distress, although this time measured through facial expression. Our results contribute to the idea that human-animal empathy shares psychological mechanisms known to mediate human-human empathy [40]. Although the PAM of empathy highlights that “similarity”, such as human appearance or phylogenetic similarity of animals, is relevant in empathetic response [2, 3], our study shows that farm animals (that are morphologically and phylogenetically dissimilar to us) still evoke emotional response in humans. The PAM of empathy underlines the automatic nature of the empathetic process, extending to our empathetic response towards animals [2]. This study expands upon the concept of overlapping neural activation in empathetic response towards humans and non-human animals [13, 41] by showing that videos of animals in pain are sufficient to elicit emotional responses measurable through facial expression.

Facial expressions provides a more accurate measure of participants’ affective states, as facial expression is inherently less controllable than other aspects of our behaviour—such as subjective ratings [18]. Although humans can control aspects of behaviour to be socially appropriate, facial expression is less susceptible to this monitoring [31]. Often, true emotion is leaked through facial expression because people are not able to see themselves perform these behaviours from the perspective of others [42]. Furthermore, facial expression plays a principle role in social communication, and facilitates an understanding of the emotion, attitudes, and ideas of others [43]. Self-assessed responses are sometimes criticised as biased, for example, by attempting to answer in a way they believe the researcher expects, or reporting in such a manner that reflects more positively on themselves [23]. The use of facial responses in our study avoids these risks associated with self-reported measures.

A secondary objective was to determine the extent to which facial expressions were related to self-reported responses. The relationship between physiological and verbal reports of emotion is cited often in the literature [3, 44, 45]. Comparison of facial expression and self-reported measures of emotional response indicates a positive relationship between these two methods of assessment. Our finding agrees with earlier work showing a relationship between self-reported measures and facial expression of negative emotions when shown human stimuli [46]. A relationship has also been shown between self-reports and physiological measures (e.g. skin conductance response, heart rate) in response to both human and animal in victimizing scenarios [3, 44, 46, 47].

Facial expression may capture the automatic motor processing of empathy [1] and self-reported responses may capture emotional attribution [44]—both of which are valuable when assessing empathetic response. Differences between physiological and self-reported measures may be due in part to social desirability confounds associated with self-report methods [12]. Facial responses may also be more sensitive to differences in attentiveness and processing [48]. The PAM of empathy states that high attention can activate stronger peripheral responses such as facial expression [1]. Our results similarly suggest that there are benefits to integrating self-report and facial expression measurements when assessing empathetic responses.

Previous studies demonstrate that people hold negative views towards painful practices [20, 49]. Urban consumers may be unaware of common farm practices [38]; previous work has shown that participants were largely unaware of procedures like castration, and largely disapproved of the practice upon learning about it [20, 38, 39]. Instead of simply describing procedures, as in typical public attitude surveys, we used publicly sourced videos to convey real-life images. Video were publicly available on YouTube, introducing variation in the animals, environment, and composition of each clip. Although the setting likely affects perceptions of the animal [35], the advantages of sourcing clips in this way is that this adds generality to our findings, and grounds our results in how these practices are performed on farms. In the few studies that previously assessed human-animal empathy, the stimulus was not necessarily the animals’ behavioral response; instead, scenes of injury, sickness, confinement or being roughly handled were used to elicit emotional responses [3, 13]. Previous research on human-human empathetic pain response have used painful medical scenarios (e.g. needle injection) with similar success [40]. This method allowed us to capture the immediate emotional reactions towards actual procedures in real-time rather than having participants respond to a verbal or written description of the procedure.

We were interested in identifying factors that influenced responses towards these painful procedures. Empathetic responses can be affected by both top-down (e.g. personal factors) and bottom up influences (e.g. the species viewed). Our study found that participants self-reported more negative emotional responses toward cow videos compared to pig videos. There is no consensus in the literature regarding human attitudes towards cows versus pigs. One study [46] found that people rated pigs higher in cognition and sentience compared to cows, but another found that people believed cows were capable of more complex emotions compared to pigs [44]. The difference in attitudes towards pigs and cows, both common food animals, may be less substantial than that towards animals that are very close to use socially (e.g. as pets) or very similar to us phylogenetically (e.g. primates) [44].

Other factors related to the video stimuli could be considered in future work. For example, animals vary in their behavioral responses during painful procedure, including body movements and vocalizations [50, 51], and these differences may affect the empathetic response. Humans draw inferences regarding distress from the facial expressions of others [52], and scientific work shows that trained human assessors can reliably assess pain in animals using facial expressions of mice and rats, among other species [52, 53 and 55 for examples]. Thus, context of the video, including whether or not facial expression of the animal is visible, may be beneficial to include in future studies.

Our study also found an effect of trait empathy on both facial expression and self-reported emotional response. The Interpersonal Reactivity Index is frequently used to assess empathy towards animals, with previous studies showing that the subscales Empathetic Concern and Personal Distress positively correlate to attitudes [6]. Previous research found that these two subscales correlated with negative attitudes towards animal cruelty [54]. Furthermore, those with high self-reported trait empathy show more intense facial reactions to human facial expression stimuli compared to those with low self-reported empathy [18, 55]. More empathetic subjects are more responsive in both somatic and self-reported aspects compared to less empathetic subjects [47]. Our results suggest that emotional response is positively correlated to trait empathy.

Other top-down processes in our study were not congruent with previous literature. For example, gender is a predictor in surveys assessing attitudes towards animals; females tend to express more concern for animal welfare than males, suggesting higher levels of empathy [35, 56, 57]. Females tend to react with more distinct facial expression responses towards other human facial expressions of emotion, and have generally shown to be more facially expressive than males [58–60]. Previous research shows that males are less likely to neuter their male dogs [61], suggesting that males may show stronger empathetic responses towards animals experiencing pain from castration. However, we found no difference between male and female participants even when examining only responses to the castration videos.

Previous research has found that decreased meat consumption is associated with increased empathy toward animals [62], but we found no evidence of a relationship between diet and emotional response. Our lack of ability to detect these demographic effects may relate to limitations of the study. Our within-subject design allowed for a strong test of the video, but the limited number of participants likely reduced our ability to meaningfully assess between subject demographic factors that have previously shown to affect facial expression, including those we tested like gender and diet (e.g. seven of eight non-omnivorous participants were female in our study), as well as others that warrant exploration, such as ethnicity or cultural background [63, 64]. We used a convenience sample of undergraduate students; these individuals cannot be considered representative of the larger population. This limitation did not introduce a confound with treatments (as these were tested within subject), but further work will be required to determine if participants from other demographics respond similarly, allowing a better understanding of demographic differences in facial expression responses of empathy. Further research should address a larger and more diverse sample to assess demographic differences in facial expression responses of empathy.

Another limitation is that the thin slice judgement method did not differentiate between different negative emotions, as judges were only asked to rate each video based on the general unpleasantness of the participants’ facial expression. The specificity of facial expressions of various emotions has been investigated. Negative emotions (e.g. pain & disgust) can be differentiated by unique muscle movements, although these overlap in their expression [15]. Our stimuli were not curated to promote “pure” pain response or “pure” disgust responses, and many videos elicited self-reported emotional responses that were a mixture of various negative emotions. Because of the range of emotions expressed, we chose to combine these when assessing facial expression using one rating of “unpleasantness”. This also allowed naïve observers to rate the videos without the extensive training required to assess specific muscle movements. More specific descriptions of facial expressions would be needed to provide a stronger basis for inferences regarding specific emotional states [45, 65]. More specificity could be achieved using an automated Facial Action Coding System program; this would overcome the training required for reliable scoring while still allowing for specificity of muscle movements for particular emotional states [65].

Another limitation was our inability to differentiate between automatic versus deliberate or feigned facial expressions. Although participants could not see the interviewer, they were told that they were being recorded (although the purpose of recording was not disclosed). It is possible that facial expressions were exaggerated or concealed for various reasons. The task given to the judges in this study did not involve the assessment of emotional leakage—when true affective state “leaks” through facial expression even when a person is trying to conceal it [17]. Untrained observers in previous studies were able to discriminate between true and false expressions at a level higher than chance, as more intense emotional responses are more difficult to conceal compared to less intense emotional responses [17]. Furthermore, our participants were explicitly instructed to think about emotional response during the interview; this has been shown to increase the true emotional response of participants [66]. Our task, combined with our stimuli being strong enough to produce measurable emotional responses, suggest that completely feigned facial expression was unlikely.

Further research in this area could use facial expression to measure empathetic responses in farm workers, as these are the people directly interacting with animals in a farm setting. More empathy towards animals is related to improved welfare of farm animals, as well as greater job satisfaction for stockpersons [5, 67], but work to date has been based only on self-reported measures. Ultimately, there is much research that can be done to aid our understanding of empathetic response towards animals, and how this affects our treatment of and interactions with them.

## 5. Conclusion

This study provides the first evidence of the validity of facial expression as a measure of emotional response towards non-human animals. Participants showed distinctive facial expressions when viewing farm animals undergoing painful management procedures. This study contributes to the growing literature suggesting a common basis to empathetic response towards human and non-human animals.

## Supporting information

S1 File Ly & Weary interview statements. Short statements used to describe farm animal management procedures to participants during the interview.
(DOCX)

S2 File Ly & Weary SAS code. Statistical analysis used to assess emotional response towards animals in pain.
(SAS)

S1 Dataset Ly & Weary emotional response dataset. Data describing scored facial expression and self-reported emotional response to videos of animals undergoing farm management procedures.
(CSV)

S2 Dataset Ly & Weary survey dataset. Data describing demographic factors of participants.
(CSV)
