# Supplementary material for: Facial expression in humans as a measure of empathy towards farm animals in pain
Source: PLoS One. 2021 Mar 1;16(3):e0247808. doi: 10.1371/journal.pone.0247808 (PMC7920373; doi:10.1371/journal.pone.0247808)
Supplement: S1 File — Short statements used to describe farm animal management procedures to participants during the interview. (DOCX) [file pone.0247808.s001.docx]

| **CD**  Calf Disbudding | Cattle naturally develop horns. To prevent horn growth, farmers often use a hot iron to burn the tissue around the horn bud. This video shows a calf being disbudded with a hot iron. |
| --- | --- |
| **CB**  Cattle Branding | Cattle are often branded using a hot iron, usually placed on the hip of the animal. This video shows a calf being branded with a hot iron. |
| **CC**  Calf Castration | Male cattle are commonly castrated, often by placing an elastic band around the scrotum to cut off blood supply. This video shows a calf being castrated with band. |
| **CR**  Calttle Restraint | For routine procedures such as vaccinations, cattle are often restrained. This video shows a cow being restrained. |
| **CN**  Cattle Neutral | Cattle spend much of their day standing. This video shows a cow standing on pasture. |
| **PD**  Piglet Tail Docking | Farmers commonly cut off the tails of piglets when they are a few days old. Tails are often cut using shears. This video shows a piglet having its tail docked. |
| **PT**  Piglet Teeth Clipping | Piglets are born with sharp, outwardly projecting teeth. Farmers often clip these teeth using shears. This video shows a piglet having its teeth clipped. |
| **PC**  Piglet Castration | Male pigs are commonly castrated, often by placing a scalpel to cut open the scrotum and severing the spermatic cords. This video shows a piglet being castrated. |
| **PN**  Piglet Neutral | Pigs use their snout to explore their environment. This video shows a piglet smelling the ground inside a barn. |
| **PR**  Piglet Restraint | For routine procedures such as vaccinations, piglets are often held. This video shows a piglet being held. |

**Interview blurbs**. Participants were shown all 10 videos described below. Participants were given one of two sets of videos.

For videos, visit <https://www.youtube.com/playlist?list=PLIb5uzAH7j-Io1zzYlfAs5r-NTdwoAuzF>
